# Comparison of Labscan 200 and FlexMap 3D Luminex for Anti‐HLA Antibodies Monitoring

**DOI:** 10.1111/tan.70731

**Published:** 2026-05-05

**Authors:** J. Milhès, L. Duclercq, S. Blavy, A. Del Bello, N. Kamar, C. Bouthemy, N. Congy‐Jolivet

**Affiliations:** ^1^ CHU Toulouse, Laboratoire d'Immunologie HLA, Institut Fédératif de Biologie Hôpital Purpan Toulouse France; ^2^ Département de Néphrologie et Transplantation d'Organe, CHU Toulouse Hôpital Rangueil Toulouse France; ^3^ Département de Biologie Vasculaire Institut Des Maladies Métaboliques et Cardiovasculaires (I2MC) Toulouse France; ^4^ Inserm UMR1291 – CNRS UMR5051 – Université Toulouse III, Institut Toulousain des Maladies Injectieuses et Inflammatoires (Infinity) Toulouse France; ^5^ CRCT, INSERM UMR 1037, Université Toulouse III Toulouse France

**Keywords:** anti‐HLA antibodies, kidney transplantation, Luminex, single antigen beads assay

## Abstract

The new Luminex model FlexMap 3D is progressively replacing the previous Luminex generation in HLA laboratories for the detection and identification of anti‐HLA antibodies. FlexMap 3D Luminex promises a higher degree of sensitivity, which may lead to greater mean fluorescence intensities, depending on the anti‐HLA beads kit supplier. Given that anti‐HLA antibodies and potential donor specific antibodies monitoring rely on MFI variations, all kits routinely used and especially Single Antigen Beads assays must be compared. In the HLA Laboratory at Toulouse Hospital, we compared the two Luminex generations using LABScreen Mix, Panel Reactive Antibody and LABScreen Single Antigen from One Lambda and Lifecodes ID from Werfen suppliers. LABScreen Mix, Panel Reactive Antibodies and Single Antigen gave the same results with both Luminex platforms. It should be noted that a divider was applied to all results obtained with FlexMap 3D when using One Lambda kits, which enabled us to obtain comparable MFI. Lifecodes ID also showed a strong correlation with both Luminex instruments, but no divider was applied and higher MFI were attained on FlexMap 3D. Serial dilutions of two monoclonal antibodies also illustrated that MFI achieved with Single Antigen from Werfen on FlexMap 3D Luminex were closer to One Lambda values. All MFI obtained with One Lambda were virtually identical when using Labscan 200 or FlexMap 3D, allowing the switch to the new Luminex platform to be made without issue. MFI gained with Werfen was higher with FlexMap 3D Luminex, which increases sensitivity, and may facilitate MFI comparisons between the two suppliers.

**Abbreviations**
Ab: antibodies
CV: coefficient of variation
cXM: cellular crossmatch
DSA: donor specific antibodies
EC50: efficient concentration at 50%
FM3D: FlexMap 3D Luminex
LRA: lowest ratio antigen
LS200: Labscan 200
MFI: mean fluorescence intensity
MoAb: monoclonal antibody
OLMIX: One Lambda LABScreen Mix
OLPRA1/OLPRA2: One Lambda LABScreen PRA Class I/II
OLSAB1/OLSAB2: One Lambda LABScreen Single Antigen Class I/II
PRA: panel reactive antibodies
SAB: single antigen beads
SFHI: Société Francophone d'Histocompatibilité et d'Immunogénétique (French Society of Histocompatibility and Immunogenetics)
SFT: Société Francophone de Transplantation (French Society of Organ Transplantation)
vXM: virtual crossmatch
WLSA1/WLSA2: Werfen Lifecodes ID Single Antigen Class I/II

## 1 Introduction

For almost 20 years, Luminex technology has become the most used biological test to screen and identify anti‐HLA antibodies (anti‐HLA Ab), notably due to its high sensitivity [1]. Every patient on the organ transplantation waiting list, as well as all transplanted patients, is monitored by Luminex anti‐HLA Ab screening and identification assays. To date, virtual crossmatches (vXM) lead the decision to perform or to omit cellular crossmatches (cXM). These vXM are essentially realised on the basis of donor and recipient HLA typing, anti‐HLA Ab Luminex results and eventual immunising events for each patient [2, 3, 4]. With graft biopsy and recent technologies like cell‐free DNA detection, anti‐HLA Ab remain a key biological analyte, especially for identifying humoral rejection when donor specific antibodies (DSA) are detected de novo after transplantation [5].

Two main suppliers provide anti‐HLA Ab screening and identification tests through Luminex technology: One Lambda and Werfen. Both use HLA antigen coated fluorescent beads to detect circulating anti‐HLA Ab and provide mean fluorescence intensities (MFI). More importantly, MFI results depend on the kit supplier, and it has been well described that One Lambda kits are most of the time more sensitive than Werfen [6, 7, 8]. Even if MFI is a semi‐quantitative value, kinetic monitoring of anti‐HLA Ab, detection of DSA and their intensity constitute a major part of post‐transplantation follow‐up. This implies that MFI monitoring should be carried out with the same kit supplier to facilitate MFI variations interpretation and to guarantee the biological significance of MFI variations when monitoring patients. However, the two suppliers are complementary and both may be used in HLA laboratories, which highlights the need for MFI equivalences between the two kit providers.

Luminex platform Labscan 200 (LS200) was released in 2005, and is still widely used in many HLA laboratories. All anti‐HLA Ab detection and identification kits are compatible with this technology, including Single Antigen Beads (SAB) assays for class I and class II given that almost 100 kinds of beads can be detected and segregated by LS200. Another Luminex platform does exist, the FlexMap 3D (FM3D) which was initially developed to increase the number of beads and the resolution of HLA typing kits (i.e., LABtype SSO, One Lambda). Replacing LS200 with the more recent model FM3D may be an interesting opportunity since acquisition is faster and supposedly more sensitive. However, this consideration raises an important question regarding MFI monitoring, as increased sensitivity may imply MFI values changes.

To our knowledge, only a few studies have investigated the concordance between LS200 and FM3D results for anti‐HLA Ab and this was limited to one kit supplier [9]; however, no one to date has compared both suppliers and platforms. We propose to compare screening and identification assays used routinely with both LS200 and FM3D Luminex. To this end, we have analysed results from One Lambda LABScreen Mix (OLMIX), One Lambda LABScreen PRA Class I/II (OLPRA1 and OLPRA2), One Lambda LABScreen Single Antigen Class I/II (OLSAB1 and OLSAB2) and Werfen Lifecodes ID Single Antigen Class I/II (WLSA1 and WLSA2).

## 2 Methods and Materials

### 2.1 Patients' Sera and Anti‐HLA Ab Profiles

Patients' sera analysed for 232 LABScreen Mix (OLMIX), 22 LABScreen PRA classes I and II (OLPRA1/2), 51 LABScreen Single Antigen Class I/II (OLSAB1/2) from One Lambda and 112 Werfen Lifecodes ID Single Antigen Class I/II (WLSA1/2) were compared. All patients were either on the kidney transplantation waiting list or had post‐kidney transplantation status. All sera were conserved at 4°C before OLMIX screening and at −20°C before identification by OLPRA1/2, OLSAB1/2 or WLSA1/2. According to French law (Loi Jardé), anonymous retrospective studies do not require Institutional Review Board approval.

Noteworthy, samples in this study comprised a wide range of OLMIX profiles, including negative, false positive, weak and strong positive profiles. Single Antigen assays (OLSAB1/2 and WLSA1/2) included various anti‐HLA profiles ranging from common anti‐HLA‐A/B/DRB1/DQB1 or anti‐Bw4/6 patterns to more specific profiles such as anti‐Cw, anti‐DP, anti‐alpha chains DQA1 and DPA1.

### 2.2 Anti‐HLA Ab Kits and Positivity Threshold

Each kit has been used according to the manufacturer's instructions. For both kits the procedures were similar: sera and beads were incubated for 30 min, followed by 30 min incubation with an anti‐IgG phycoerythrin conjugate, then acquisition immediately performed after beads resuspension. The main difference relies on beads to serum ratios. For all One Lambda kits (OLMIX, OLPRA1/2 and OLSAB1/2), the ratio was 1:4 (5 μL beads and 20 μL serum) whereas it was 4:1 (40 μL beads and 10 μL serum) for WLSA1/2 kits. Additionally, according to SFT‐SFHI recommendations and previously published data [10, 11], EDTA (final concentration 0.05 M) was added to sera to avoid complement prozone effect in OLSAB1/2 and WLSA1/2 assays.

Anti‐HLA Ab screening (OLMIX) was assessed by LABScreen Mixed Class I & II Antibody Screening (LSM12, lot 025, One Lambda, Canoga Park, USA, delivered by Thermofisher, Waltham, Massachusetts, USA). Each experiment included the LABScreen Negative Control (LSNC) which allowed us to precisely define the following thresholds. Negative thresholds were 2.2 and 3.0‐fold LSNC raw value for classes I and II respectively; positive thresholds were 2.8 and 3.5‐fold LSNC raw value respectively. These thresholds were characterised by the laboratory for each new batch according to the manufacturer's instructions. For OLPRA1/2, respectively LABScreen Panel Reactive Antibody (PRA) classes I (LS1PRA lot 021) and II (LS2PRA, lot 020) (One Lambda), the thresholds depended on the negative control raw value. It should be noted that PRA interpretation takes into consideration the profile rather than the strict MFI.

Anti‐HLA Ab identification was performed with LABScreen SAB classes I and II (One Lambda) (OLSAB1/2) (LS1A04, lot 015 for class I, LS2A01, lot 016 for class II) and Lifecodes ID classes I and II (Werfen, Barcelona, Spain) (WLSA1/2) (265,100, lot 3,014,507 for class I; 265,200, lot 3,014,513 for class II). For OLSAB1/2, positivity threshold was 1000 for baseline MFI values, defined as follows: (sample raw MFI bead—sample raw MFI negative bead)—(negative control raw MFI bead—negative control raw MFI negative bead). For WLSA1/2, positivity threshold was 750 for raw value MFI. Negative and positive controls were included in each Single Antigen experiment (LSNC and a homemade positive control for OLSAB1/2; negative and positive controls included in the kits for WLSA1/2). Background noise for OLSAB1/2 and MFI/LRA ratio for WLSA1/2 were taken into account for positive or negative assignment. For MFI values normalisation on each bead, MFI was divided by the RAD (relative antigen density) for WLSA1/2, and by the MFI obtained with the monoclonal antibodies W6/32 (for class I) and FJ (for class II) for OLSAB1/2. RAD, W6‐32 and FJ MFI were provided in the technical worksheets of each kit supplier for the corresponding lot.

### 2.3 Luminex Platforms and Interpretation Softwares

Each experiment was conducted using both Luminex platforms (Luminex, Genk, Belgium): LABScan 200 (LS200), and FlexMap 3D (FM3D). For every comparison presented in this manuscript, each experiment was performed once and acquired on LS200 then FM3D consecutively, to minimise inter‐assay variability. Minimum count for each bead was reached for every sample (50 for One Lambda kits, 60 for Werfen). Sixteen patients' sera were analysed using both suppliers' SAB kits (OLSAB1/2 and WLSA1/2), and the data presented come from only two distinct experiments (one plate for each kit supplier). Data acquisition from all experiments was carried out by xPonent software V.4.3 (Luminex). All One Lambda kits results were interpreted by HLA Fusion software V.4.6.1 while all Werfen kits results were interpreted by Match It software V.1.5.2.2.

It should be noted that data generated on FM3D with One Lambda kits were mathematically modified, with the application of a divider (quotient 1.67) according to manufacturer's specifications. All data acquired on LS200 for both kits' suppliers and for WLSA1/2 on FM3D did not undergo this division.

### 2.4 Monoclonal Antibodies Directed Against HLA Classes I and II

Two chimeric monoclonal antibodies specific to HLA class I public epitopes for HLA‐A, HLA‐B and HLA‐C (Anti‐HLA class I control, CL1‐42‐01, human IgG1κ constant and murine W6/32 MoAb variable regions, Invivogen, Toulouse, France) and to HLA class II public epitopes for HLA‐DR, HLA‐DP and HLA‐DQ2 (Anti‐HLA class II control, CL2‐42‐01, human IgG1κ constant and murine F3.3 MoAb variable regions, Invivogen) [12] were serially diluted (4‐fold dilution from 8.0 to 4.9 × 10^−4^ μg/mL) in PBS with 6% bovine serum albumin. The two first dilutions (for OLMIX and OLSAB1/2) or only the first one (for WLSA1/2) gave maximum MFI (signal saturation), whereas the two last dilutions tended towards negativity with both kit suppliers. The same serial dilution has been used for all experiments, and the same plate was acquired on both Luminex platforms consecutively. The mean of all class I or all class II beads was calculated to compare OLMIX results. For OLSAB1/2 and WLSA1/2 results, the mean of all class I beads and the mean of HLA‐DR, HLA‐DP and HLA‐DQ2 for class II, was also calculated for each kit supplier and each platform.

### 2.5 Statistical Analysis

All sigmoid curves were determined with Boltzmann non‐linear regression. Boltzmann non‐linear regression curves also gave the effective concentration at 50% (EC50). Wilcoxon matched pairs signed‐rank test was used to compare two sigmoid curves. Spearman test confirmed that the pairing was effective.

To compare more than two sigmoid curves, non‐parametric Anova comparison with Friedman test was applied. Dunn's multiple comparison test then compared each pair of sigmoid curves.

For all correlation graphs between LS200 and FM3D MFI, linear regression gave slope and *R* ^2^ for lines fit and Spearman correlation (*r*s) was also assessed. Cohen's kappa index [13] was used to estimate concordance between the two platforms for OLMIX.

All statistical analyses were performed using GraphPad Prism 9.5.1 software.

## 3 Results

### 3.1 Labscan 200 and FlexMap 3D Acquisitions Were Highly Concordant for OLMIX and OLPRA1/2

**FIGURE 1:**
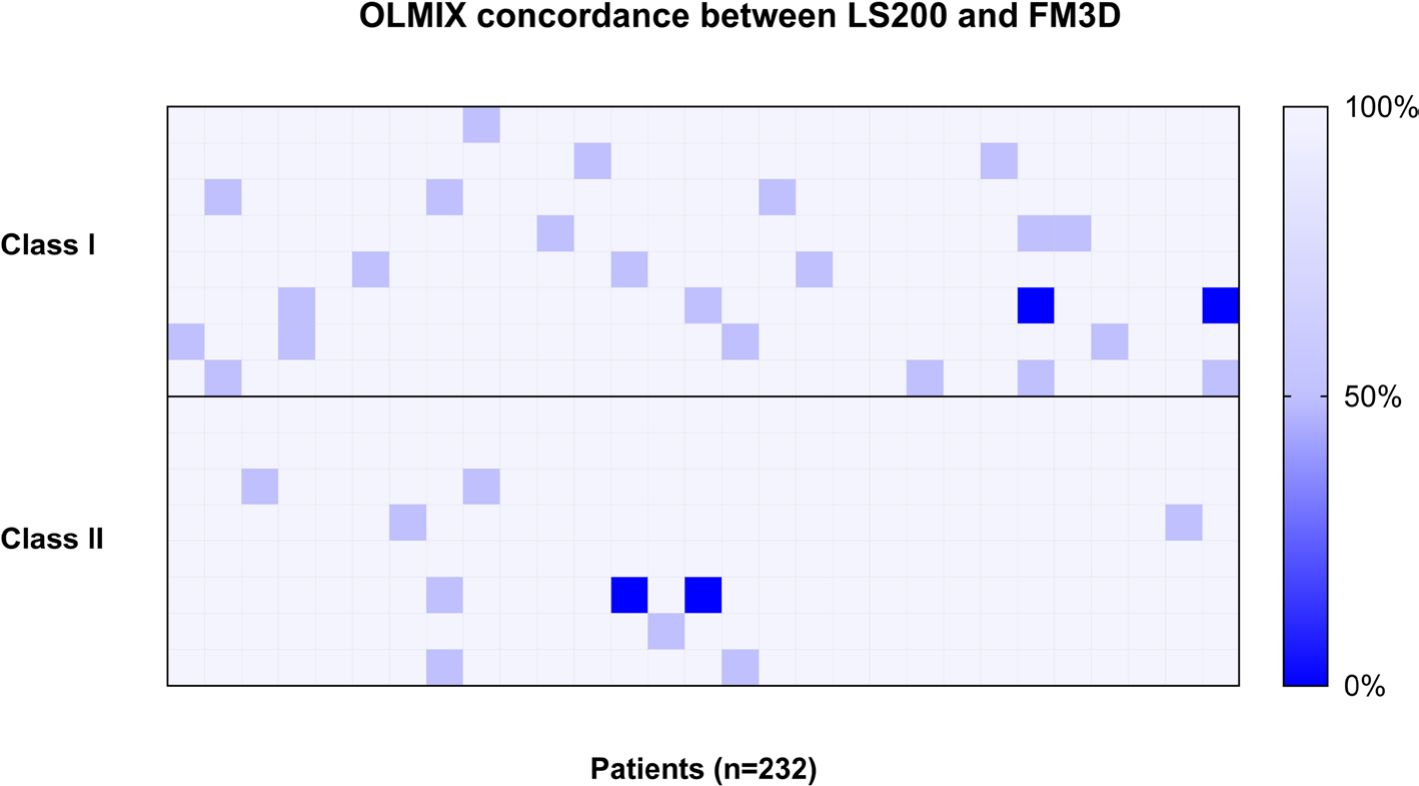
Qualitative comparison of LABScreen Mix (OLMIX) between LS200 and FM3D. Fusion software assignment (negative/undetermined/positive) was compared for *n* = 232 patients' sera including negative, false positive and positive profiles. The concordance between LS200 and FM3D acquisitions is represented by the following colours: 100% (white squares) indicated identical assignment between LS200 and FM3D; 50% (pale purple) denoted a slight difference (from positive to undetermined, or undetermined to negative), and 0% (dark blue) portrayed a major discrepancy (from positive to negative). Each square corresponds to the concordance between both Luminex devices for each patient's sera, for either class I (up) or class II (down). Concordance was 89.7% for class I and 96.1% for class II and the weighted Cohen's kappa index was 0.87 for class I and 0.92 for class II. Thirty‐four discordances were observed between both platforms. 23/24 discordances for class I and 9/10 for class II originated from undetermined results on LS200 which became negative on FM3D. Only one sample was discordant for both classes I and II.

Three LABScreen Mix (OLMIX) routine experiments were acquired with both Luminex platforms (LS200 and FM3D). Two hundred and thirty‐two anti‐HLA Ab profiles were compared. For each profile, HLA Fusion assigned negativity, positivity or ‘undetermined’ result, depending on the thresholds defined in our laboratory. Figure 1 shows the concordance between Fusion software assignment for LS200 and FM3D as a heatmap (concordance: 89.7% for class I and 96.1% for class II). The weighted Cohen's kappa index was 0.87 for class I and 0.92 for class II. Discordances only concerned anti‐HLA Ab profiles close to the thresholds: expected positive and negative assignments were correctly identified on both devices. Fusion software assignment produced less undetermined results on FM3D than LS200. After interpretation of Fusion assignment and correction by HLA biologists, concordance was determined to be 100% for both classes I and II.

Serial dilutions of two monoclonal antibodies (MoAb) specific to HLA public epitopes were tested with OLMIX. The means of classes I and II beads MFI were calculated for each dilution and each platform. For class I (Figure S1a) and class II (Figure S1b), the two curves perfectly fitted except for the highest MFI values, which were slightly higher on FM3D than LS200. As a consequence, EC50 on FM3D was slightly increased compared to LS200, even if EC50 for class I and class II was similar on both Luminex platforms.

Two LABScreen PRA class I (OLPRA1, 13 samples) and two PRA class II (OLPRA2, 9 samples) experiments were acquired on both Luminex platforms. Linear regression produced very good correlations for OLPRA1 (slope = 0.94, *p* < 0.0001, Spearman *r*s = 0.98, *p* < 0.0001) (Figure S2a) and OLPRA2 (slope = 0.93, *p* < 0.0001, Spearman *r*s = 0.99, *p* < 0.0001) (Figure S2b). Coefficients of variation (CV) of each bead baseline MFI between LS200 and FM3D were calculated and were generally below 35% (OLPRA1, 56 beads) or 20% (OLPRA2, 35 beads) (data not shown). The beads exhibiting the highest MFI baseline variations were negative on both platforms and assignment concordance determined by HLA biologists remained 100%.

### 3.2 The Divider Guaranteed MFI Homology for OLSAB1/2, Whereas Its Absence for WLSA1/2 Conferred Higher MFI on FM3D

**FIGURE 2:**
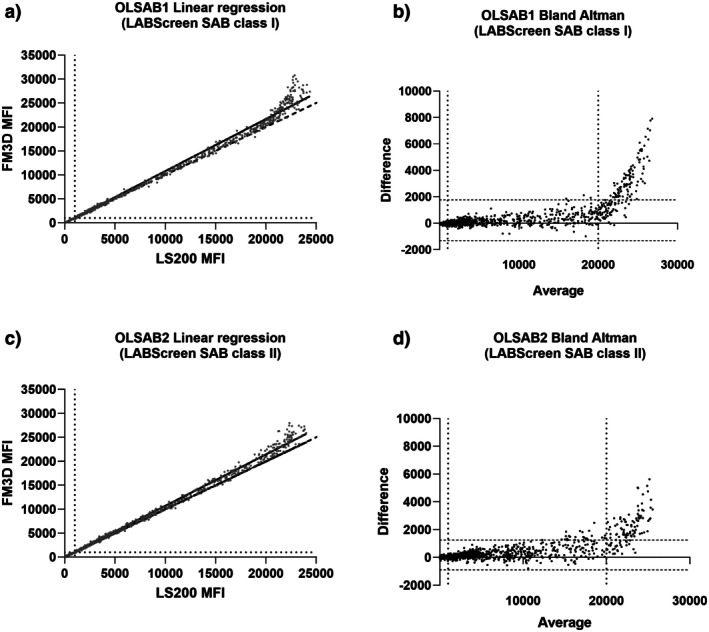
Quantitative comparison of LABScreen single antigen classes I and II (OLSAB1/2) between LS200 and FM3D: (a, c) are linear regression for all baseline MFI values obtained for classes I (a) and II (c) between LS200 and FM3D. Dot lines represents line of identity (*y* = *x*) and point lines depict thresholds (1000 MFI); (b, d) are the Bland–Altman representations of the difference between each measurement, depending on the mean (average) for each bead. The two vertical dot lines correspond to 1000 and 20,000 MFI. The two horizontal dot lines denote the 95% limits of agreement between the two platforms. For note, since baseline MFI obtained with OLSAB1/2 kits may be equal to zero for negative beads, all MFI under 1.0 were artificially increased to 1.0. (a) Regression line for OLSAB1. *N* = 26 patients' sera and 2522 beads. FM3D MFI ranged from 1.0 to 30,774 (mean = 3791, standard deviation = 7069, first quartile = 2.0, third quartile = 3153), whereas LS200 MFI ranged from 1.0 to 24,361 (mean = 3566, standard deviation = 6481, first quartile = 2.0, third quartile = 3015). Equation line: MFI (FM3D) = 1.09 × MFI (LS200) (*p* < 0.0001; *R* ^2^ > 0.99). Spearman *r*s = 0.996, *p* < 0.0001. (b) Bland–Altman representation for OLSAB1: Difference between LS200 and FM3D MFI, depending on MFI average of both platforms. Ninety‐five percent limits of agreement were −1314; 1766 (horizontal dotted lines). Above 20,000 MFI (vertical dotted lines), the systematic bias depending on MFI increased (21.9 for MFI < 10,000, 489 for 10,000 < MFI < 20,000, 2620 for MFI > 20,000). (c) Regression line for OLSAB2. *N* = 25 patients' sera and 2375 beads. FM3D MFI ranged from 1.0 to 27,973 (mean = 3159, standard deviation = 6197, first quartile = 6.0, third quartile = 2903), whereas LS200 MFI ranged from 1.0 to 23,990 (mean = 2979, standard deviation = 5765, first quartile = 1.0, third quartile = 2748). Equation line: MFI (FM3D) = 1.07 × MFI (LS200) (*p* < 0.0001; *R* ^2^ > 0.99). Spearman *r*s = 0.993, *p* < 0.0001. (d) Bland–Altman representation for OLSAB2: Difference between LS200 and FM3D MFI depending on MFI average of both platforms. Ninety‐five percent limits of agreement were −892.5; 1251 (horizontal dotted lines). Above 20,000 MFI (vertical dot lines), the systematic bias depending on MFI increased (46.5 for MFI < 10,000, 652 for 10,000 < MFI < 20,000, 2115 for MFI > 20,000).

Anti‐HLA Ab identifications with One Lambda LABScreen SAB kits classes I (OLSAB1, 26 samples) and II (OLSAB2, 25 samples) were performed on both platforms. Concerning class I, a significant correlation was observed between LS200 and FM3D MFI (slope = 1.09, Spearman *r*s = 0.99, *p* < 0.0001). However, there was a difference for high MFI (maximum MFI = 24,361 for LS200, 30,774 for FM3D) suggesting an absence of linearity between both platforms for MFI above 20,000 (Figure 2a). The Bland–Altman representation of the difference depending on each bead mean MFI confirmed the homology of MFI under 20,000, given that most of these points were within the 95% limits of agreement whereas MFI above 20,000 fell outside of the 95% agreement and systematic bias dramatically increased (Figure 2b). Coefficients of variation for all beads MFI were compared between LS200 and FM3D and showed a few variations (Figure S3a).

Concerning class II, a significant correlation between the two Luminex platforms was also obtained (slope = 1.07, *p* < 0.0001, Spearman *r*s = 0.99, *p* < 0.0001). As for class I, high MFI tended to increase faster on FM3D, which explains the difference between maximum MFI measured (23,990 for LS200, 27,973 for FM3D) (Figure 2c). The Bland–Altman representation for class II beads showed homology for most MFI under 20,000 whereas a large majority of MFI above 20,000 were not within the 95% limits of agreement (Figure 2d). Moreover, the systematic bias depending on MFI confirmed this observation. A few variations between LS200 and FM3D MFI were also noted as most of the CV were under 15% (Figure S3b). Finally, OLSAB1 and OLSAB2 MFI quantitative comparison on both Luminex platforms depicted an almost identical homology for MFI under 20,000, then higher MFI on FM3D than LS200 for MFI above 20,000.

**FIGURE 3:**
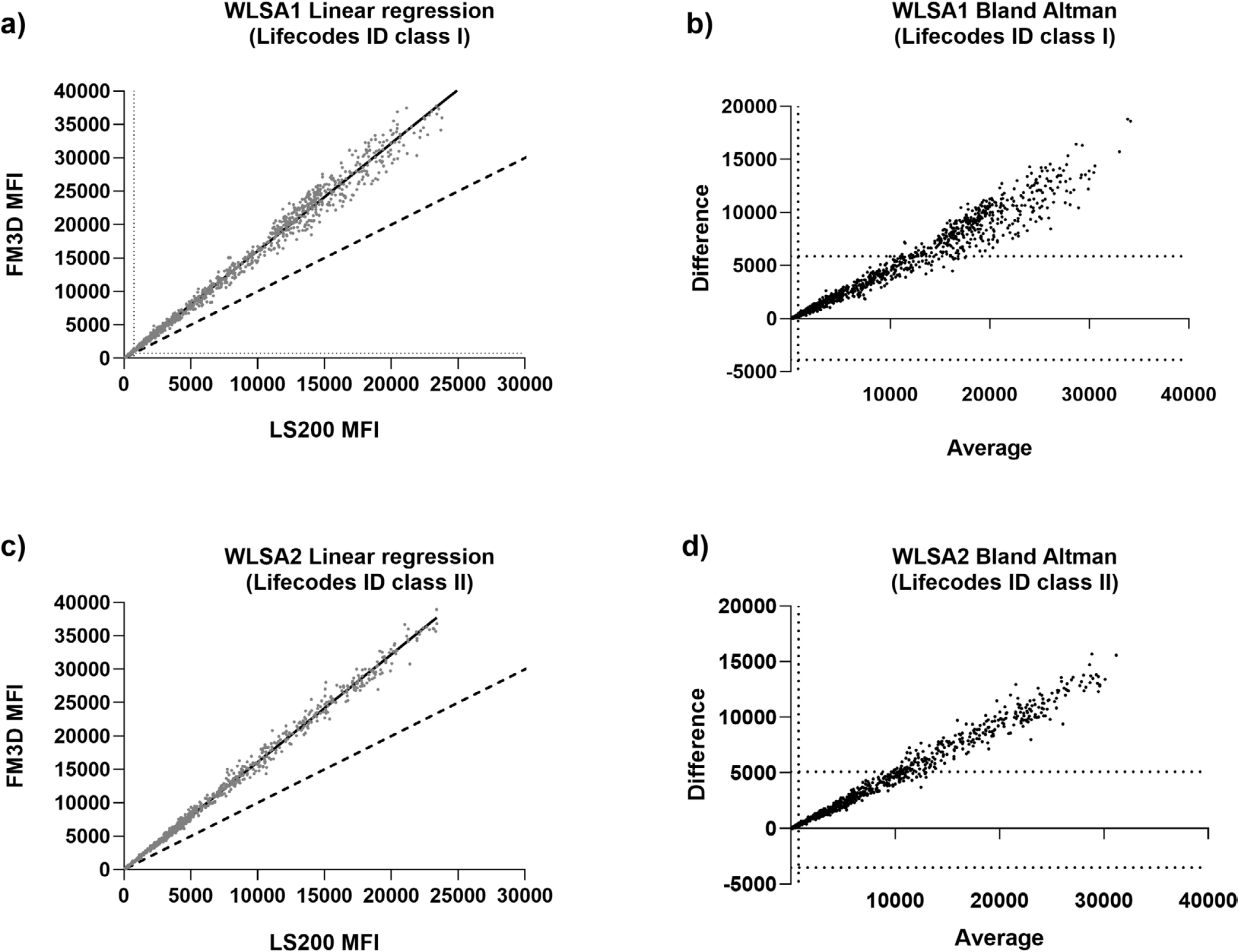
Quantitative comparison of Lifecodes ID classes I and II (WLSA1/2) between LS200 and FM3D: (a, c) are linear regressions for all MFI raw values obtained for classes I (a) and II (c) between LS200 and FM3D; (b, d) represent the Bland–Altman representations of the difference between each measurement depending on the mean (average) for each bead. The vertical dotted line represents 750 MFI cut‐off. The two horizontal dotted lines represent the 95% limits of agreement between the two platforms. (a) Regression line for WLSA1. *N* = 62 patients' sera and 5952 beads. FM3D MFI ranged from 42 to 43,434 (mean = 2649, standard deviation = 6465, first quartile = 186, third quartile = 872), whereas LS200 MFI ranged from 25 to 25,145 (mean = 1672, standard deviation = 4003, first quartile = 139, third quartile = 571). Equation line: MFI (FM3D) = 1.61 × MFI (LS200) (*p* < 0.0001; *R* ^2^ > 0.99). Dot lines denote line of identity (*y* = *x*). Spearman *r*s = 0.97, *p* < 0.0001. (b) Bland–Altman representation for WLSA1: Difference between LS200 and FM3D MFI depending on the MFI average of both platforms. Ninety‐five percent limits of agreement were −3902; 5855 (horizontal dotted lines). The systematic bias dramatically increased with MFI (213 for MFI < 5000, 6300 for 5000 < MFI < 15,000, 10,927 for MFI > 15,000). (c) Regression line for WLSA2. *N* = 50 patients' sera and 4800 beads. FM3D MFI ranged from 16 to 38,965 (mean = 2133, standard deviation = 5739, first quartile = 91, third quartile = 472), whereas LS200 MFI ranged from 8.0 to 23,416 (mean = 1340, standard deviation = 3555, first quartile = 82, third quartile = 293). Equation line: MFI (FM3D) = 1.61 × MFI (LS200) (*p* < 0.0001; *R* ^2^ > 0.99). Dot lines denote line of identity (*y* = *x*). Spearman *r*s = 0.92, *p* < 0.0001. (d) Bland–Altman representation for WLSA2: Difference between LS200 and FM3D MFI depending on the MFI average of both platforms. Ninety‐five percent limits of agreement were −3510; 5095 (horizontal dotted lines). The systematic bias dramatically increased with MFI (198 for MFI < 5000, 5614 for 5000 < MFI < 15,000, 11,036 for MFI > 15,000).

Anti‐HLA Ab identifications with Werfen Lifecodes ID Single Antigen kit class I (WLSA1, 62 samples) and class II (WLSA2, 50 samples) were performed. Once again, each experiment was acquired on LS200 and FM3D (both with no MFI divider). For WLSA1, a high correlation between LS200 and FM3D MFI was observed (slope = 1.61, *p* < 0.0001, Spearman *r*s = 0.97, *p* < 0.0001) (Figure 3a). The 95% limits of agreement of Bland–Altman representation were wide and contained less than half of the points. Indeed, for MFI > 10,000, most of the measured differences fell outside of the upper limit of agreement and the systematic bias dramatically increased since MFI were higher than 5000 (Figure 3b).

For WLSA2 a strong correlation was also obtained between both platforms (slope = 1.61, *p* < 0.0001, Spearman *r*s = 0.92, *p* < 0.0001) (Figure 3c). The 95% limits of agreement of Bland–Altman representation were similar to class I and most of the points were outside of the upper limit while the systematic bias also dramatically increased with MFI (Figure 3d). Likewise, this representation confirmed that FM3D produced higher MFI than LS200 although this increase was proportional to MFI. Finally, each bead CV between both platforms was calculated; given that all CV were between 20% and 50%, Figure S3c,d established that the MFI variations arose mainly due to a constant increase in FM3D MFI versus LS200 MFI. The absence of divider clearly conferred higher MFI on FM3D and the increase in MFI on FM3D was proportional to LS200 MFI.

### 3.3 LS200 and FM3D Luminex Gave Identical Results With Neat and Diluted Sera From Hyper‐Sensitised Patients

As performing serum dilution may be necessary, particularly to more precisely evaluate the amount of circulating anti‐HLA Ab, the effect of 1:10 dilution on MFI obtained with both Luminex platforms was assessed. Five hyper‐sensitised patients' sera were analysed on LS200 and FM3D, undiluted or after 1:10 dilution with OLSAB1 and OLSAB2 kits, according to guidelines for imlifidase eligibility [14]. Only four discordances were observed after comparison of all profiles obtained with neat and diluted sera (Figure S4). With the defined threshold, 3 beads were positive on LS200 and negative on FM3D, and the contrary for one bead. Calculated PRA according to Eurotransplant virtual PRA calculator [15] was estimated for neat and diluted sera on both Luminex platforms. Only one sample (Patient A) exhibited a 0.10% variation of calculated PRA with neat serum and no variation on calculated PRA after dilution. No variation of cPRA was observed for the 4 other samples, with neat nor diluted sera. Finally, 9/10 cPRA were identical between LS200 and FM3D while only one serum presented a 0.10% variation, which would not have any impact on patient eligibility for protocol nor anti‐HLA Ab delisting on allocation programme (cPRA = 98.57% with LS200, and 98.47% with FM3D).

### 3.4 MFI Equivalence Between OLSAB1/2 and WLSA1/2 With Both Platforms

The serial dilution of the two anti‐HLA MoAb was analysed with the two SAB kit suppliers and both Luminex platforms. Boltzmann non‐linear regression curves and EC50 allowed us to compare these four conditions for classes I and II.

**FIGURE 4:**
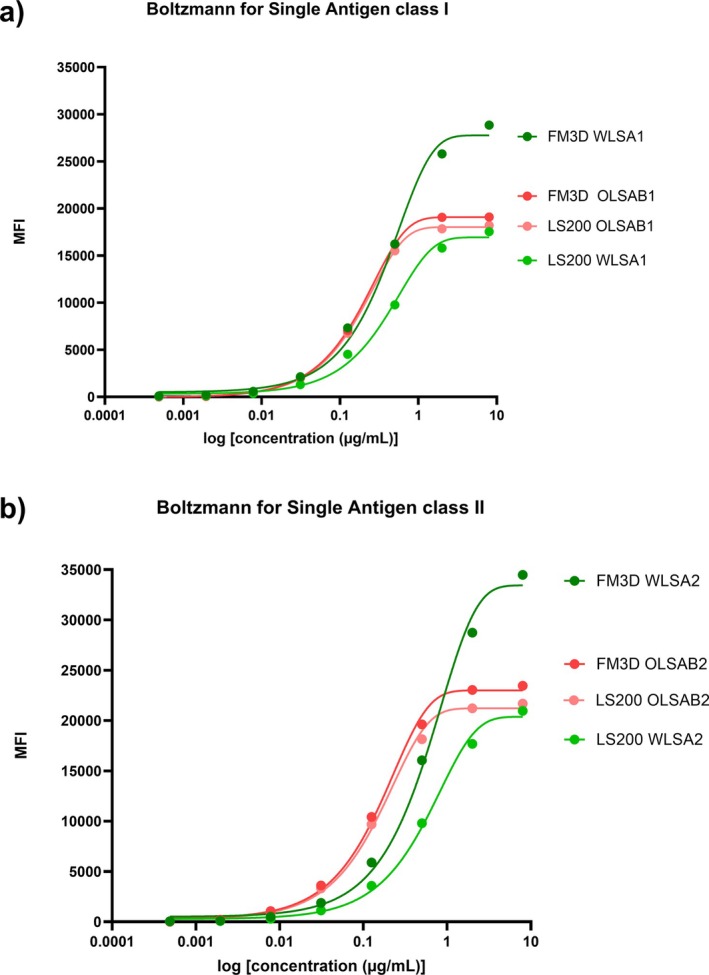
Comparison of OLSAB1/2 and WLSA1/2: MFI amplitude and EC50: (a, b) Two monoclonal antibodies pan‐specific for class I, and DR, DP and DQ2 HLA antigens respectively, were serially diluted, and incubated with OLSAB1/2 and WLSA1/2 beads, then analysed with LS200 and FM3D. Boltzmann and least squared regressions gave the same EC50 value, and *R* ^2^ > 0.99 for all non‐linear regressions. (a) The four curves represent the Boltzmann non‐linear regression of the mean of all class I beads for each test (all *R* ^2^ > 0.99). The highest MFI measured on LS200 and FM3D for OLSAB1 were close (18,346 and 19,404 MFI respectively), as was the case for LS200 WLSA1 maximum MFI (18,652). WLSA1 gave a higher maximum MFI on FM3D (30,585). EC50 were similar between LS200 and FM3D for each supplier: 0.175 and 0.179 μg/mL with OLSAB1, 0.424 and 0.421 μg/mL with WLSA1 respectively. Friedman test (non‐parametric Anova comparison) illustrated that the four curves were significantly different (*p* = 0.0003). However, post hoc analysis with Dunn's multiple comparisons test revealed that LS200 and FM3D WLSA1 were significantly different (*p* = 0.0006), as was the case for FM3D WLSA1 and LS200 OLSAB1 (*p* = 0.003). Interestingly, FM3D WLSA1 and FM3D OLSAB1 were not significantly different (*p* = 0.32), and as expected LS200 and FM3D OLSAB1 did not differ (*p* = 0.73). Threshold recalculation (bottom of the Boltzmann sigmoid plus 2 or 3 s): 932 and 1285 for LS200 OLSAB1, 927 and 1328 for FM3D OLSAB1, 657 and 958 for LS200 WLSA1, 817 and 1184 for FM3D WLSA1. (b) The four curves represent the Boltzmann non‐linear regression of the mean of all DR, DP and DQ2 class II beads for each test (all *R* ^2^ > 0.99). As the monoclonal anti‐HLA class II antibody does not recognise all HLA specificities, all negative beads (DQ3, DQ4, DQ5 and DQ6 specificities) were removed for mean MFI calculation. The highest MFI measured on LS200 and FM3D for OLSAB2 were close (22,042 and 23,895 MFI respectively), as was the case for LS200 WLSA2 maximum MFI (22,376). WLSA2 gave a higher maximum MFI on FM3D (37,008). EC50 were similar between LS200 and FM3D for each supplier: 0.149 and 0.150 μg/mL with OLSAB1, 0.621 and 0.635 μg/mL with WLSA1 respectively. Friedman test (non‐parametric Anova comparison) illustrated that the four curves were significantly different (*p* = 0.006). Post hoc analysis with Dunn's multiple comparisons test revealed the only statistically significant difference was between LS200 WLSA2 and FM3D OLSAB2 (*p* = 0.003). Threshold recalculation (bottom of the Boltzmann sigmoid plus 2 or 3 s): 708 and 981 for LS200 OLSAB2, 858 and 1187 for FM3D OLSAB2, 480 and 665 for LS200 WLSA2, 603 and 830 for FM3D WLSA2.

OLSAB1 and OLSAB2 curves perfectly fitted on both platforms, even if maximum MFI was slightly increased on FM3D. Interestingly, LS200 WLSA1 and WLSA2 curves did not fit with OLSAB1 and OLSAB2 curves although they reached almost the same maximum MFI. As expected, since FM3D did not apply the 1.67 divider, WLSA1 and WLSA2 reached much higher MFI on FM3D than OLSAB1/2 on both platforms and WLSA1/2 on LS200 (Figure 4a,b). For each supplier and for class I and II respectively, EC50 values were very similar between LS200 and FM3D. FM3D WLSA1 Boltzmann sigmoid almost fitted with both OLSAB1 sigmoid curves for MFI ranging from 100 to 15,000 (Figure 4a). Indeed, Friedman test showed that the four conditions were significantly different (*p* = 0.0003) but Dunn's multiple comparison revealed that LS200 and FM3D OLSAB1 were not different (*p* = 0.73), and most interestingly, FM3D WLSA1 and FM3D OLSAB1 were also not significantly different (*p* = 0.32). In contrast, the FM3D WLSA1 curve was significantly different from LS200 WLSA1 (*p* = 0.0006) and LS200 OLSAB1 (*p* = 0.003). For class II, FM3D WLSA2 MFI tended towards both OLSAB2 sigmoid curves, although with no overlay between the curves (Figure 4b). Moreover, Friedman test showed again that the four conditions were significantly different (*p* = 0.006) and Dunn's multiple comparison only revealed a significant difference between LS200 WLSA2 and FM3D OLSAB2 (*p* = 0.003).

Boltzmann sigmoid parameters were used to virtually recalculate the thresholds for the two kit suppliers on both platforms: bottom of the sigmoid plus two or three standard deviations of the residues gave quite the same thresholds for OLSAB1 and OLSAB2. As expected, WLSA1 and WLSA2 threshold values on LS200 were weaker than OLSAB1/2 thresholds. Interestingly, for class I WLSA1 thresholds were higher on FM3D and closer to OLSAB1 and OLSAB2 thresholds. This reinforces the observation of an almost overlay between FM3D WLSA1 and OLSAB1 curves (Figure 4a). On the other hand, for class II WLSA2 thresholds were also higher on FM3D than LS200 ones but still inferior to the ones measured with OLSAB2. Again, this reinforces the observation that FM3D WLSA2 curve did not overlay with OLSAB2 curves on Figure 4b.

Equivalences between LS200 and FM3D MFI were estimated. To this end, the MFI from 16 samples analysed for both classes and both kit suppliers were compared (*n* = 1392 beads for class I, *n* = 1104 beads for class II). Several MFI levels were defined for LS200 WLSA1 and WLSA2, with a particular interest for 500 (grey zone threshold), 750 (positivity threshold), 3000 and 8000 (moderate and high MFI thresholds) and 20,000 (maximum MFI on LS200). MFI equivalences were extrapolated for these LS200 MFI levels, with both suppliers on FM3D (Table 1). Fundamentally, MFI equivalences for WLSA1 and WLSA2 for clearly negative beads on LS200 (MFI < 500) did not reach positivity threshold and stayed clearly negative on FM3D. When the positivity threshold was exceeded on LS200, all MFI on FM3D were clearly positive. As expected, OLSAB1 and OLSAB2 MFI were higher than WLSA1 and WLSA2 up to 10,000 MFI on LS200. However, after 10,000, MFI became higher on WLSA1 and WLSA2 kits (and attained more than 30,000 maximum MFI) than OLSAB1 and OLSAB2 (maximum MFI for OLSAB1 were under 25,000) (Figure S5a,c). When considering the grey zone threshold on LS200 (MFI comprised between 500 and 750), equivalences on FM3D showed very similar values for the two suppliers and both classes. Indeed, most MFI between 500 and 750 on LS200 were found to be positive on FM3D for WLSA1 and WLSA2, and at least in grey zone for OLSAB1 and OLSAB2 on FM3D. For class I, OLSAB1 MFI clearly increased faster than WLSA1 after 500 MFI on LS200 (Figure S5b) whereas WLSA2 and OLSAB2 equivalences for low MFI almost overlapped on FM3D (Figure S5d).

**TABLE 1:** Proposal MFI equivalence between LS200 and FM3D for OLSAB1/2 and WLSA1/2.

LS200	FM3D			
WLSA1/2	WLSA1	WLSA2	OLSAB1	OLSAB2
250	390	460	140	420
350	540	630	300	460
**500**	**780**	**870**	**580**	**680**
**750**	**1120**	**1260**	**1630**	**1150**
850	1270	1400	1700	1300
1000	1500	1570	1950	1470
1500	2200	2480	3150	2500
2000	3050	3260	4000	3500
**3000**	**4700**	**4600**	**6400**	**5500**
4000	6350	6350	8500	7500
5000	7400	8400	9800	9500
6500	10,250	10,250	12,350	10,750
**8000**	**12,000**	**12,600**	**13,300**	**12,500**
10,000	15,650	17,400	15,750	14,000
12,000	18,000	18,500	17,500	14,500
15,000	23,650	24,500	20,000	18,250
18,000	27,900	29,600	22,000	20,100
**20,000**	**30,800**	**31,000**	**22,000**	**21,000**
25,000	34,250	33,700	24,500	22,000

*Note:* Sixteen patients' samples were analysed on WLSA1/2 and OLSAB1/2 kits as well as on LS200 and FM3D. For every defined MFI level on LS200 WLSA1/2 (left column), the corresponding MFI for each kit on FM3D were calculated (30 beads under and above, i.e., 61 beads for each MFI level). For the lasts ranges, only 55 and 51 (for 20,000 MFI, class I and II) and 31 (for 25,000 MFI) beads were used to calculate the means. MFI close to the positivity threshold (500, 750), moderate and high positivity (3000 and 6000) and saturation signal (20,000) on LS200 are indicated in bold.

Lastly, the performances of individual beads were assessed with the data generated after serial dilution of the two MoAb (cf Figure 4): all data are presented in Figure S6. Briefly, a significant spread of maximum MFI was observed, with considerable disparities between all beads. Some outlier beads were identified; they were largely the same among each kit supplier between LS200 and FM3D. Above all, huge disparities between HLA loci appeared, which prompted us to represent beads MFI mean for each locus. A substantial spread of maximum MFI was also observed (Figure S7). For class I, HLA‐A and HLA‐B beads had close maximum MFI, but HLA‐C was systematically under the former's values. For class II, we also observed that HLA‐DR and HLA‐DP had similar maximum MFI, while HLA‐DQ2 was below. Recalculated threshold, EC50 and maximum MFI for each locus and each condition are summarised in Table S1. Interestingly, when normalised by each bead relative antigenic density (RAD), maximum MFI dispersion was lowered for WLSA1/2 kits. As HLA‐C beads display a lower antigenic density than HLA‐A and HLA‐B, most of normalised MFI values for HLA‐C beads became higher than HLA‐A and HLA‐B ones. For class II, dispersion was also attenuated, although some beads showed increased normalised MFI compared to the three loci (DRB1*12:02/11:01/11:03/11:04). In the absence of antigenic density for OLSAB1/2 kits, normalisation of MFI was performed by the MoAb W6/32 (for class I) and FJ (for class II) MFI. For both class I and class II, normalisation did not reduce the dispersion of maximum MFI, especially for class I (data not shown). No difference between LS200 and FM3D was observed concerning normalised MFI for both kit suppliers.

## 4 Discussion

Anti‐HLA antibodies screening and identification allow the declaration of forbidden HLA antigens before transplantation, monitoring of patients on organ waiting list—especially after immunising events—and follow‐up of transplanted patients. These assays are also indispensable to establish virtual crossmatch [2, 3]. Each HLA laboratory can adopt its own strategy to manage anti‐HLA Ab follow‐up [16] in line with the official recommendations [17]; the worldwide assay is currently still Single Antigen Bead testing by Luminex. Most of the time, HLA laboratories use one of the two main kit suppliers: One Lambda or Werfen. For many years, some discordances have been described between the two kits, especially for SAB [18] and several publications have tried to highlight the reasons for these discordances: antibodies targeting denatured HLA molecules [19, 20, 21, 22], prozone effects [23, 24], variations in epitope structure leading to sensitivity and specificity differences [25]. To elucidate these discordances, the comparison of both SAB assays results and the comparability with other techniques (i.e., cellular crossmatches) may help the interpretation. Given the consequences of this interpretation, all the information has to be considered before giving a definitive result. Therefore, when the time comes to change Luminex platform or kit supplier, all precautions must be taken to guarantee the best consistency as possible in rendering anti‐HLA Ab results.

During this study, all results obtained from One Lambda kits were perfectly comparable between both Luminex platforms. This was mainly due to the application of a 1.67 quotient divider for all MFI acquired with FM3D. Indeed, the results obtained with OLMIX were 100% concordant after biologist interpretation for every sample tested. The same concordance was observed with OLPRA1 and OLPRA2, then OLSAB1 and OLSAB2 with linear regression slopes close to 1.0 for all these assays. Anti‐HLA Ab Luminex reproducibility is weak, and calculated coefficients of variation are often > 10% when comparing identical samples on the same platform [12]. The CV acquired between the two Luminex results were under 35% (except for a few beads), and the majority under 15%, which confirmed the concordance of the results achieved with both platforms. It should be noted that with OLSAB1 and OLSAB2 the MFI increase produced by FM3D acquisition was counterbalanced by the 1.67 divider for MFI ranging from 0 to 20,000. However, higher MFI than 20,000 were obtained with FM3D than LS200. Indeed, maximum MFI can now reach 30,000 with OLSAB1, and 25,000 with OLSAB2. Comparison of anti‐HLA Ab profiles with undiluted and 1:10 diluted sera revealed negligible consequences on calculated PRA. This is particularly relevant for HLA laboratories performing screening to ascertain eligibility for imlifidase injection, which includes anti‐HLA Ab testing with neat and 1:10 diluted sera, as serum dilution results determine anti‐HLA Ab delisting for kidney allocation in this protocol [14]. For WLSA1/2 kits, the absence of divider with FM3D acquisition obviously led to an increase for all MFI compared to LS200. However, the correlation remained excellent between LS200 and FM3D MFI because linear regression showed that FM3D MFI were 1.61 times higher than LS200 MFI on both WLSA1 and WLSA2 assays. Interestingly, these 1.61 slopes were very close to the 1.67 divider applied for OLSAB1/2 kits, and to the 1.67 value measured in a previous study [9]. As a result, the maximum MFI measured on FM3D with WLSA1 and WLSA2 assays rose to 35,000 MFI for both classes I and II.

EC50 obtained with dilutions of anti‐HLA MoAb allowed us to objectively compare beads' affinity for both kit suppliers and fluorescence acquisition by both Luminex platforms [12, 26]. As EC50 were similar between LS200 and FM3D for each supplier and both classes I and II assays, replacement of the Luminex platform did not impact SAB kits' performances. An explanation can be found among the following reasons: for OLSAB1 and OLSAB2 kits, the 1.67 divider allowed an almost identical homology between all MFI and therefore, the EC50 was not impacted. For WLSA1 and WLSA2, all MFI were higher with FM3D; lowest and maximum MFI were equally affected and the EC50 of the sigmoid curves did not vary. As expected, LS200 WLSA1 and WLSA2 curves were different from OLSAB1 and OLSAB2 ones, with lower MFI. Notably, FM3D WLSA1 and WLSA2 curves were closer to OLSAB1 and OLSAB2 ones, leading to an almost identical superposition for FM3D WLSA1 and OLSAB1 curves. Nevertheless, FM3D did not allow for an exact superposition of One Lambda and Werfen MFI, especially for WLSA2. EC50 were still higher with WLSA1/2 kits than OLSAB1/2 and MFI was still not equivalent between the two kits.

No huge difference was noted between calculated thresholds on LS200 and FM3D for OLSAB1 and OLSAB2; all calculated thresholds around 850 and 1100 MFI fell close to the 1000 MFI positivity threshold considered for this assay. On the contrary, LS200 and FM3D thresholds for WLSA1 and WLSA2 were different. For LS200, class I positivity threshold (800 MFI) stood around the manufacturer's one (750 MFI) and were even lower for class II (570 MFI). With FM3D, calculated thresholds were 1000 and 800 MFI respectively: FM3D seemed to increase WLSA1 and WLSA2 MFI, leading to upper thresholds and a better homology between the two kit suppliers. Given that Werfen's MFI are known to be lower than One Lambda values on LS200 [27, 28], we wanted to explore and estimate these MFI differences on FM3D with patients' sera. First, all WLSA1 and WLSA2 MFI found negative on LS200 (under 500 MFI grey zone threshold) were also negative with OLSAB1 and OLSAB2 kits and FM3D WLSA1 and WLSA2. For MFI between 500 and 750 on LS200, the increase in Werfen's MFI on FM3D conferred MFI closer to One Lambda's values. Interestingly, equivalences for class I and class II were different for these low MFI: WLSA1 and OLSAB1 MFI were comparable under 500 MFI although OLSAB1 MFI increased faster (especially after positivity threshold). For class II, WLSA2 and OLSAB2 were comparable up to 1500 MFI, which includes grey zone and positivity threshold. Then, One Lambda's MFI were higher than Werfen's ones until 15,000 (for class I) or 10,000 (for class II). For high MFI, WLSA1 and WLSA2 MFI increased faster than OLSAB1 and OLSAB2 and reached higher maximum MFI. Globally, OLSAB1/2 MFI were still higher than WLSA1/2 values, but FM3D tended to lower the difference between the two kit suppliers, especially for MFI close to the thresholds.

Essentially, all the calculations presented (MFI estimations, thresholds) have only been used as tools to compare kit suppliers and Luminex platforms. All these values must not be considered as a proposition of new thresholds for anti‐HLA Ab Single Antigen Bead assays. All the comparisons established between LS200 and FM3D could lead to slightly different quotients or linear regression slopes in other HLA laboratories, given the intrinsic variability of MFI measurement, Luminex lasers settings and many other parameters. Moreover, as we and others have shown, patients, locus and sometimes even individual beads have their own thresholds [27]. Overall, the values we obtained for these thresholds were largely similar to the positivity and grey zone thresholds routinely used with both suppliers.

## 5 Conclusions

In conclusion, replacing LS200 with the FM3D model does not drastically modify thresholds, MFI and biologist interpretation. For all One Lambda kits, systematic application of the 1.67 divider for FM3D MFI guaranteed an almost identical homology with LS200 MFI. For Werfen kits, MFI obtained with FM3D were 1.61 times higher than LS200 MFI. Moreover, the lower MFI obtained on LS200 with WLSA1/2 kits compared to OLSAB1/2 kits was partially attenuated and FM3D conferred a better homology between the two kit suppliers. Lastly, LS200 can be replaced by FM3D Luminex with no risk to the patient, no change in MFI interpretation for OLMIX, OLPRA/12 and OLSAB1/2 kits, but minor modifications for WLSA1/2 assays.

## Author Contributions

All the authors contributed substantially to this study. L. Duclercq and S. Blavy realised all the experiments and data acquisition. A. Del Bello and N. Kamar are the physicians in charge of the patient's follow‐up and proofread the manuscript. C. Bouthemy, N. Congy‐Jolivet and J. Milhès are the HLA biologists who designed this research study and proofread the manuscript. N. Congy‐Jolivet also contributed to manuscript redaction. J. Milhès performed the study, collected and analysed data and wrote the paper.

## Funding

The authors have nothing to report.

## Ethics Statement

According to French law (Loi Jardé), anonymous retrospective studies do not require Institutional Review Board approval.

## Conflicts of Interest

The authors declare no conflicts of interest.

## Supporting information


**Supplementary S1** Supplementary captions for figures and table.

**Figure S1:** LS200 and FM3D fluorescence acquisitions were similar.

**Figure S2:** Comparison of OLPRA1/2 between LS200 and FM3D.

**Figure S3:** Bead variations between LS200 and FM3D for all SAB assays.

**Figure S4:** LS200 or FM3D Luminex did not affect serum dilution results.

**Figure S5:** Curve representation of MFI equivalences between LS200 WLSA1/2 MFI and FM3D MFI for OLSAB1/2 and WLSA1/2.

**Figure S6:** Individual beads MFI for all conditions and outliers.

**Figure S7:** Loci means MFI for all conditions.

**Data S1:** Supporting Information.

**Table S1:** Threshold, EC50 and maximum MFI by locus for all conditions.

## Data Availability

Data are available on request due to privacy/ethical restrictions. The data that support the findings of this study are available on request from the corresponding author, J. Milhès.
