# Supplementary material for: Comparison of Labscan 200 and FlexMap 3D Luminex for Anti‐HLA Antibodies Monitoring
Source: HLA. 2026 May 5;107:e70731. doi: 10.1111/tan.70731 (PMC13144444; doi:10.1111/tan.70731)
Supplement: Supplementary file 1 — Supplementary S1 Supplementary captions for figures and table. [file TAN-107-e70731-s007.docx]

**Supplementary Figure 1: LS200 and FM3D fluorescence acquisitions were similar**

Two monoclonal antibodies (pan-specific for class I and specific for DR, DP and DQ2 HLA antigens respectively) were serial diluted and incubated with OLMIX. For class I and II respectively, all beads MFI values were close, allowing us to calculate the mean of all class I or class II MFI beads for each dilution.

(a) The two curves represent the Boltzmann non-linear regression of the mean for all 12 HLA class I beads MFI values, analyzed with LS200 and FM3D (R²>0.99). The highest MFI value was slightly higher on FM3D (23716) than LS200 (22687). Boltzmann and least squared regressions gave the same EC50 value, and R²>0.99 for both non-linear regressions. EC50 were 0.072µg/mL for LS200 and 0.080µg/mL for FM3D. Wilcoxon matched pairs signed rank test showed the two curves were not statistically different (p=0.375) after verification that pairing was significantly effective (Spearman rs=0.96, p=0.0014).

(b) The two curves represent the Boltzmann non-linear regression of the mean for all 5 HLA class II beads MFI values, analyzed with LS200 and FM3D (R²>0.99). The highest MFI value was slightly higher on FM3D (26073) than LS200 (23348). Boltzmann and least squared regressions gave the same EC50 value, and R²>0.99 for both non-linear regressions. EC50 are 0.039µg/mL for LS200 and 0.049µg/mL for FM3D. Wilcoxon matched pairs signed rank test showed the two curves were not statistically different (p=0.938) after verification that pairing was significantly effective (Spearman rs=1.00, p=0.0002).

**Supplementary Figure 2: Comparison of OLPRA1/2 between LS200 and FM3D**

Comparison of class I (n=13 patients’ sera) and class II (n=9 patient’s sera) PRA demonstrated a very good correlation between both Luminex platforms.

(a) Linear regression for all baseline MFI values obtained with class I PRA between LS200 and FM3D. Equation line: MFI (FM3D) = 0.938 x MFI (LS200) (p<0.0001, R²>0.99). N=728 MFI values. Spearman rs=0.98, p<0.0001. When compared bead by bead, coefficient of variation (CV) between LS200 and FM3D baseline MFI showed 64.7% (471/728 beads) under 15%, and 31.3% (228/728 beads) between 15% and 35%.

(b) Linear regression for all baseline MFI values obtained with class II PRA between LS200 and FM3D. Equation line: MFI (FM3D) = 0.931 x MFI (LS200) (p<0.0001, R²>0.99). N=315 MFI values. Spearman rs=0.99, p<0.0001. When compared bead by bead, CV between LS200 and FM3D baseline MFI showed 57.4% (181/315 beads) under 15% and 41.6% (131/315 beads) between 15% and 35%.

**Supplementary Figure 3: Bead variations between LS200 and FM3D for all SAB assays**

Coefficient of variation (CV) for class I (a, c) and class II (b, d) MFI values for OLSAB1/2 (a, b) and WLSA1/2 (c, d) between LS200 and FM3D. For note, since baseline MFI obtained with OLSAB1/2 kits may be equal to zero for negative beads, all MFI under 1.0 were artificially increased to 1.0. As a consequence, 0.0001 were added to all CV to avoid CV values of 0.0 for two beads with the same baseline (*i.e.* 1.0 on LS200 and FM3D).

(a) A huge majority of CV were under 15% variation, and only 158/2522 (6.3%) CV were above 30%. Most of these 158 CV above 30% concerned low baseline MFI: LS200 MFI above 100 were only found for 11/158 (7.0%), and among them only 4/158 (2.5%) had MFI above 200 (and under 500). The other beads (143/158) involved baseline values under 100.

(b) The majority of CV were under 15% variation, and only 235/2375 (9.9%) CV were above 30%. All of these 235 CV above 30% pertained to low baseline MFI: 6/235 (1.3%) were between 100 and 200 MFI, 3/235 (2.6%) were between 200 and 500 MFI, whereas the others (96.1%, 226/235) related to MFI under 100.

(c) CV under 20% were obtained for the lowest MFI (MFI < 500). Thereafter, the huge majority of CV were between 20% and 50%. 35% (2063/5952) of the measured CV were above 30%. Among these 2063 beads, 53% (1103/2063) had LS200 MFI under 500, 15% (307/2063) had LS200 MFI between 500 and 1500, 17% (341/2063) had LS200 MFI between 1500 and 10000, and 15% (313/2063) had LS200 MFI above 10000.

(d) CV under 20% were obtained for the lowest MFI (essentially under 500). Thenceforth, most of the CV were between 20% and 50%. 43% of the measured CV (2080/4800) were above 30%. Among these 2080 CV, 65% (1344/2080) had LS200 MFI under 500, 9% (187/2080) had LS200 MFI between 500 and 1500, 17% (359/2080) had LS200 MFI between 1500 and 10000, and 9% (190/2080) had LS200 MFI above 10000.

**Supplementary Figure 4: LS200 or FM3D Luminex did not affect serum dilution results**

Five hyper-sensitized patients were analyzed on both Luminex platforms with OLSAB1 and OLSAB2 kits. All MFI obtained with pure (a) and 1:10 diluted (b) sera are represented as heatmaps. Low MFI are depicted in white and high MFI are denoted in dark blue. For each patient, the whole anti-HLA Ab profile on LS200 then FM3D is represented with pure serum as well as the same profile after 1:10 dilution.

**Supplementary Figure 5: Curve representation of MFI equivalences between LS200 WLSA1/2 MFI and FM3D MFI for OLSAB1/2 and WLSA1/2**

MFI equivalence for class I (a and b) and II (c and d). For each LS200 WLSA1/2 MFI level, the mean and standard deviation for 61 beads were calculated (except for the lasts ranges: only 55 and 51 (for 20000 MFI, class I and II) and 31 (for 25000 MFI) beads were used). Each point represents the mean, and error bars are standard error of the mean (SEM).

(a) All MFI equivalences for class I. From 100 to 10000 on LS200, MFI were slightly higher on OLSAB1. After 10000 MFI, WLSA1 reached higher MFI than OLSAB1.

(b) Focus on MFI equivalences close to the threshold for class I. Most of MFI under 500 on LS200 stayed negative on both Single Antigen class I kits and WLSA1 MFI were slightly higher than OLSAB1 for these weak MFI. After 500 on LS200, most MFI turned positive on FM3D for both kits and OLSAB1 MFI were higher than WLSA1 ones.

(c) All MFI equivalences for class II. From 100 to 10000 on LS200, OLSAB2 and WLSA2 MFI almost overlapped. After 10000 MFI, WLSA2 attained higher MFI than OLSAB2.

(d) Focus on MFI equivalences close to the thresholds for class II. Most of MFI under 500 on LS200 remained negative on FM3D and similar MFI were observed with both Single Antigen class II kits.

**Supplementary Figure 6: Individual beads MFI for all conditions and outliers**

Individual sigmoid for each bead analyzed after monoclonal antibodies serial dilution. All the curves represent individual MFI for all HLA class I (HLA-A in red, HLA-B in green, HLA-C in blue) (a, c, e, g) and class II (HLA-DR in red, HLA-DQ2 in green, HLA-DP in blue) (b, d, f, h).

OLSAB1/2 (a, b, e, f) and WLSA1/2 (c, d, g, h) analyzed with LS200 (a – d) and FM3D (e – h). Some outliers have been highlighted when their maximum MFI were lower than MFI displayed by the entire locus: A69, A*24:02 DR8, DP15, and DPB1*13:01 / DPA1*03:01 for OLSAB1/2 and A69, B46, B48 and DRB1*13:05 for WLSA1/2.

**Supplementary Figure 7: Loci means MFI for all conditions**

Boltzmann sigmoid in relation to both Luminex platforms for each class I (a, b) and class II (c, d) locus on OLSAB1/2 (a, c) and WLSA1/2 (b, d). Disparities between loci were similar between both kit suppliers, with lower maximum MFI for locus HLA-C and HLA-DQ2 on LS200 and FM3D.

**Supplementary Table 1: Threshold, EC50 and maximum MFI by locus for all conditions**

For Single Antigen classes I and II kits, threshold, EC50 and maximum MFI are presented after non-linear Boltzmann regression, locus by locus. Thresholds are estimated as bottom of the Boltzmann sigmoid plus three standard deviations.
