# Supplementary figures and images for: Comparison of Labscan 200 and FlexMap 3D Luminex for Anti‐HLA Antibodies Monitoring
Source: HLA. 2026 May 5;107:e70731. doi: 10.1111/tan.70731 (PMC13144444; doi:10.1111/tan.70731)

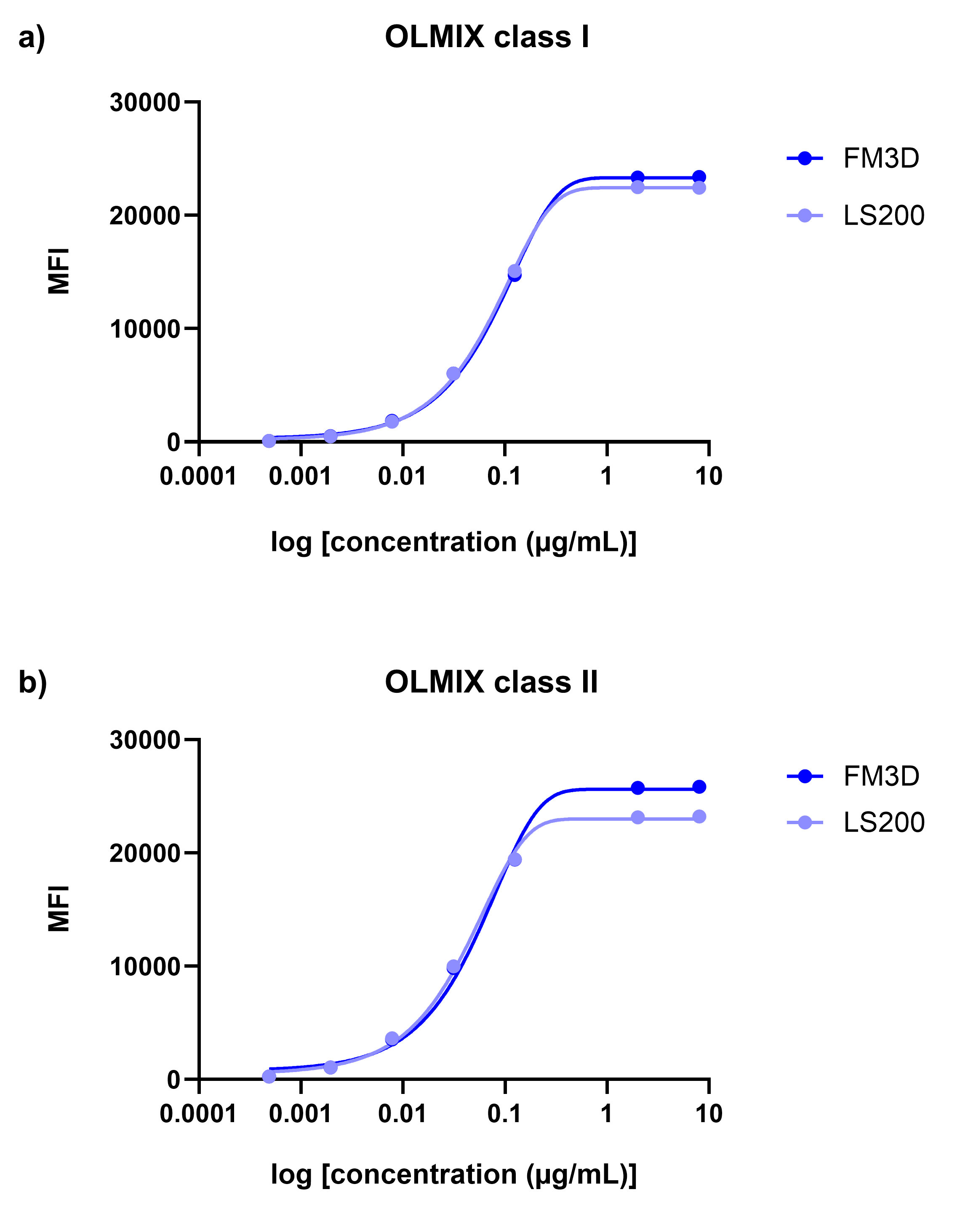

Supplement: Supplementary file 2 — Figure S1: LS200 and FM3D fluorescence acquisitions were similar. [file TAN-107-e70731-s003.tif]

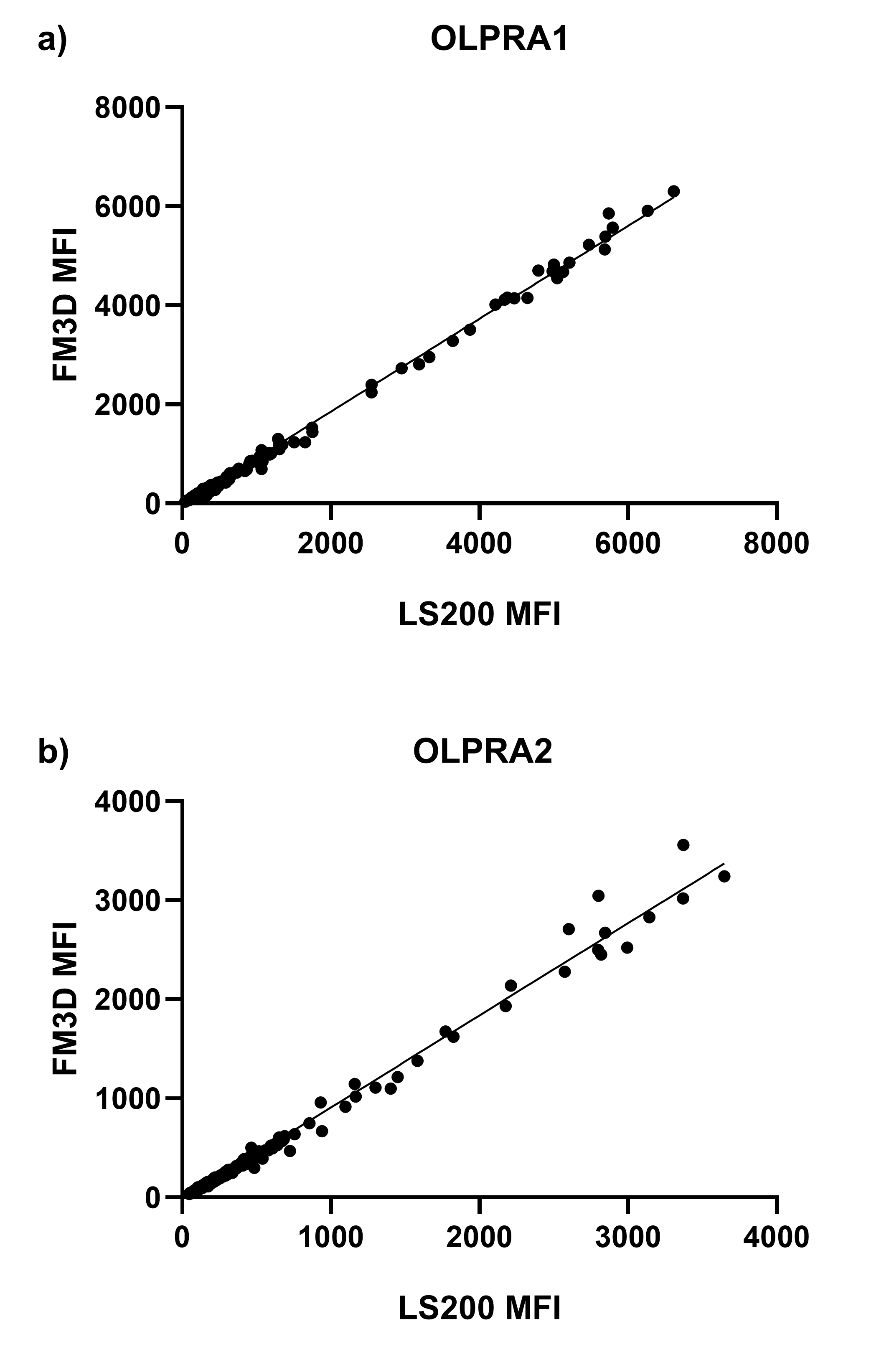

Supplement: Supplementary file 3 — Figure S2: Comparison of OLPRA1/2 between LS200 and FM3D. [file TAN-107-e70731-s009.tif]

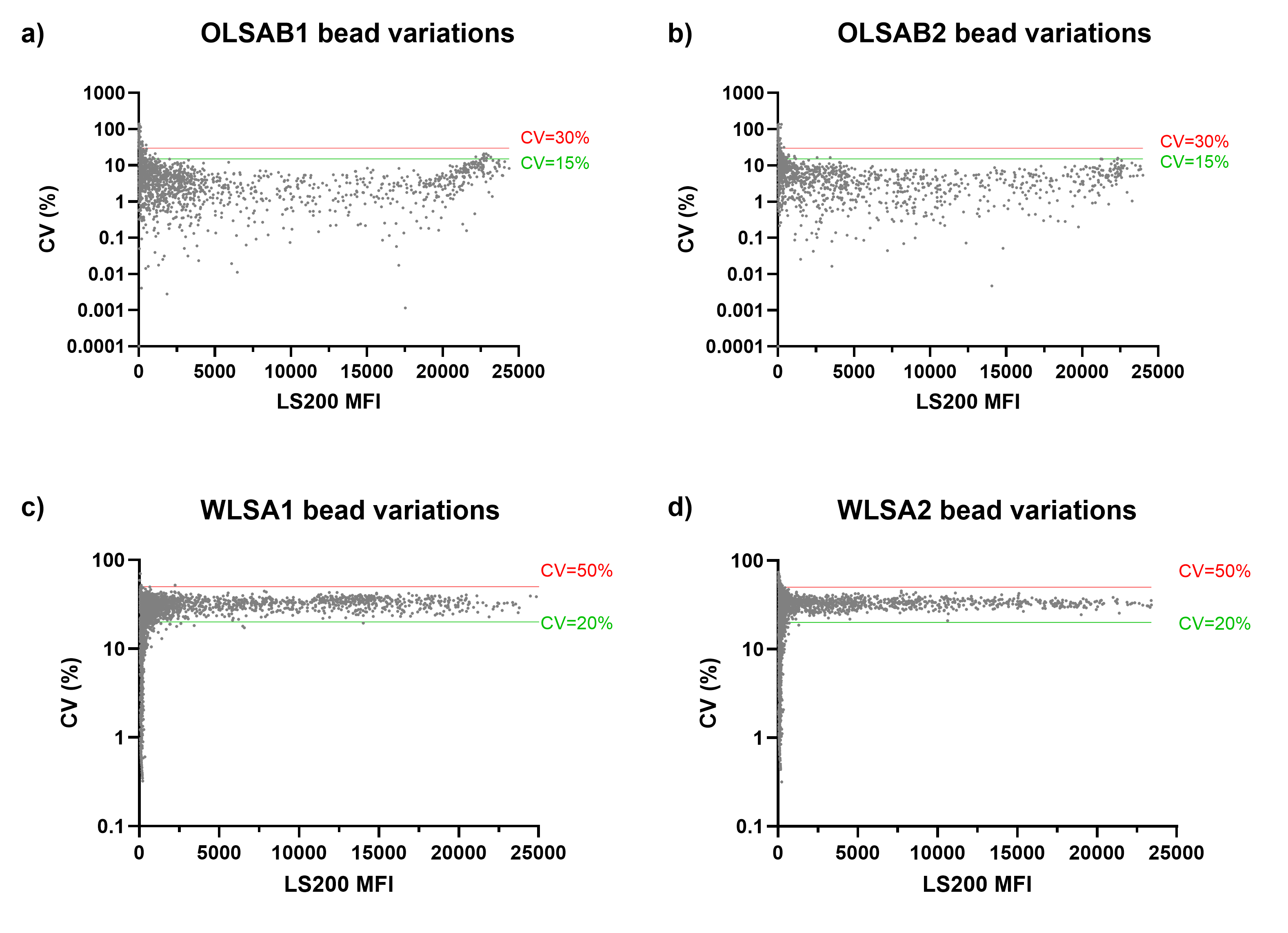

Supplement: Supplementary file 4 — Figure S3: Bead variations between LS200 and FM3D for all SAB assays. [file TAN-107-e70731-s006.tif]

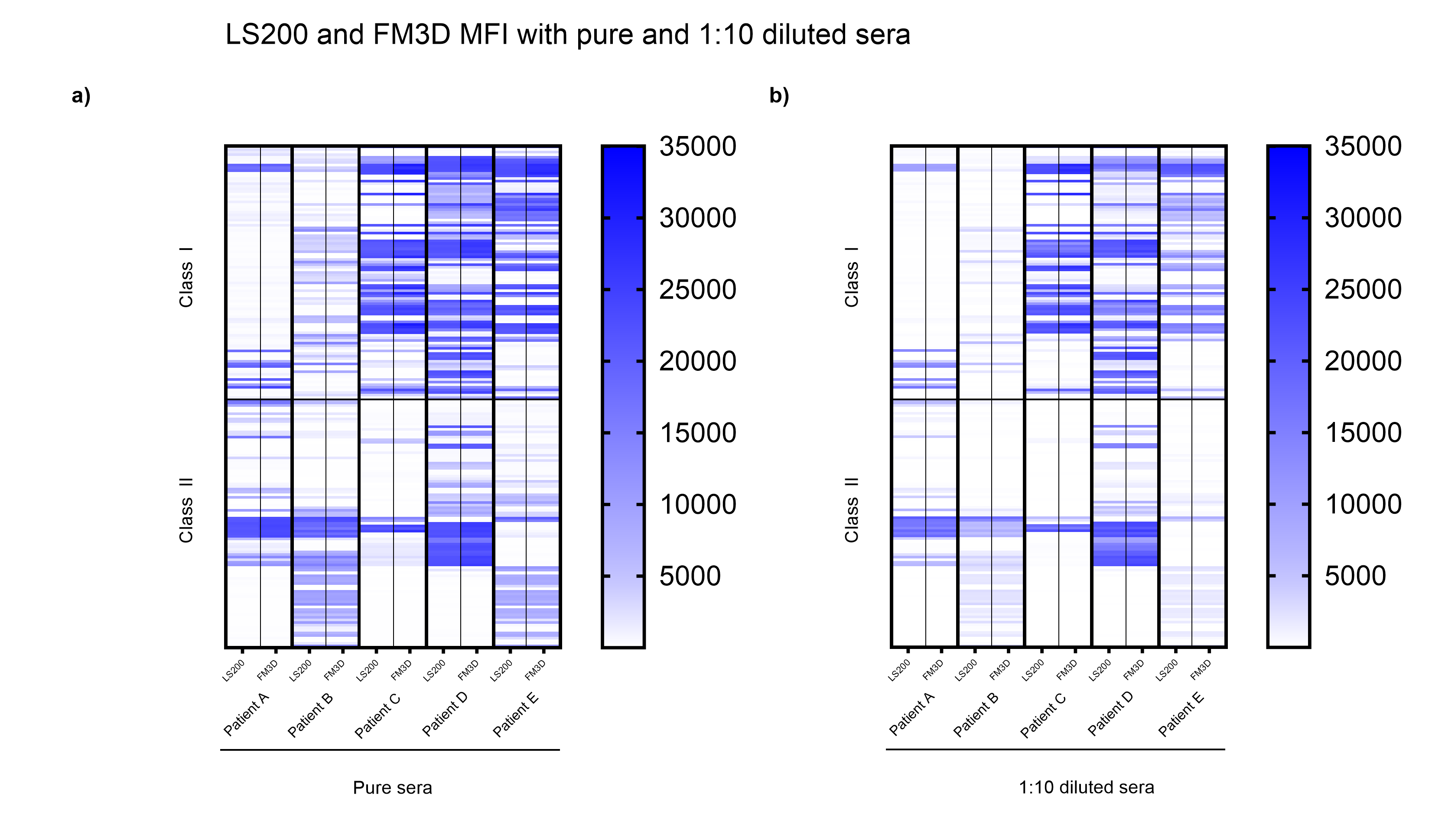

Supplement: Supplementary file 5 — Figure S4: LS200 or FM3D Luminex did not affect serum dilution results. [file TAN-107-e70731-s010.tif]

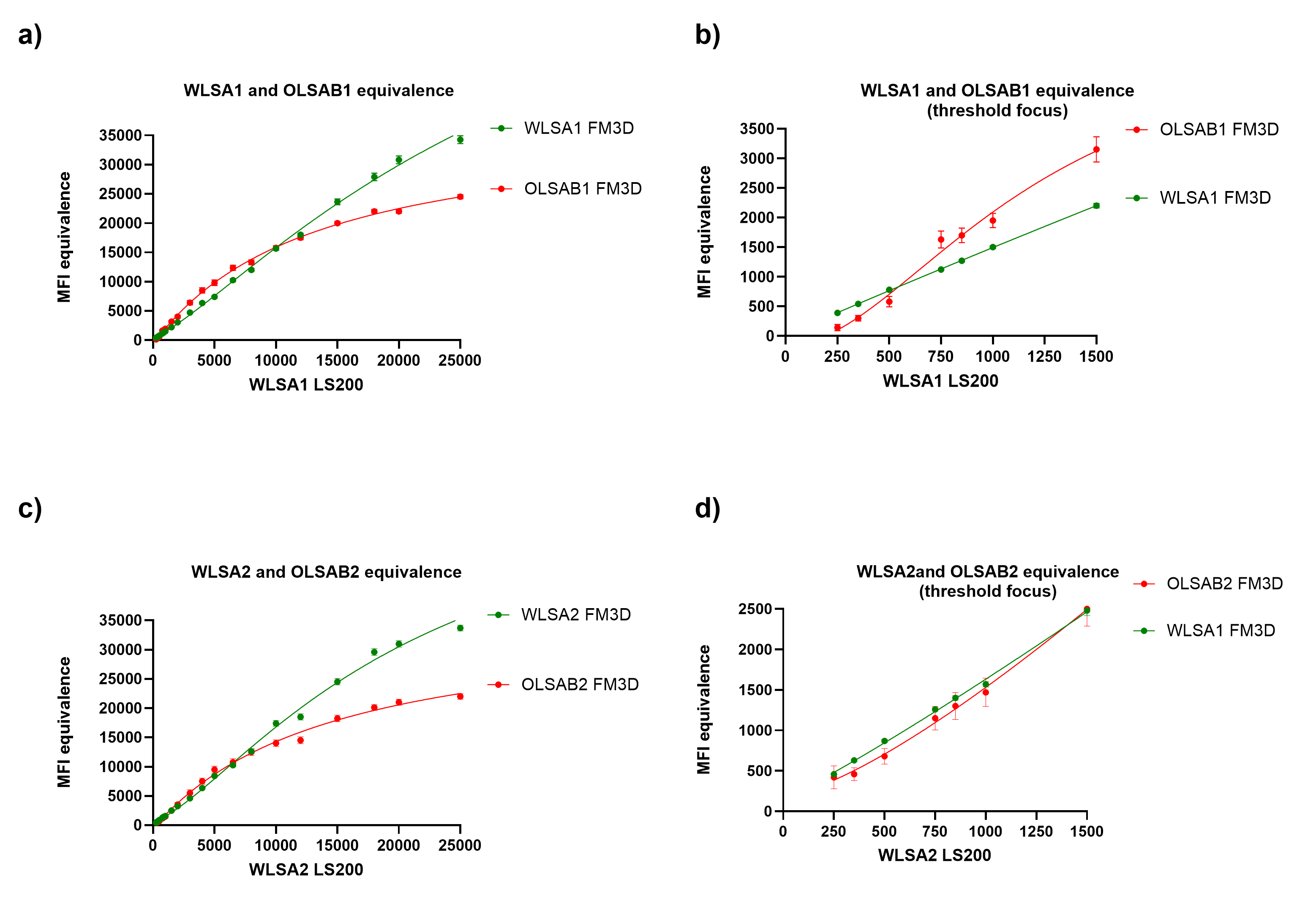

Supplement: Supplementary file 6 — Figure S5: Curve representation of MFI equivalences between LS200 WLSA1/2 MFI and FM3D MFI for OLSAB1/2 and WLSA1/2. [file TAN-107-e70731-s008.tif]

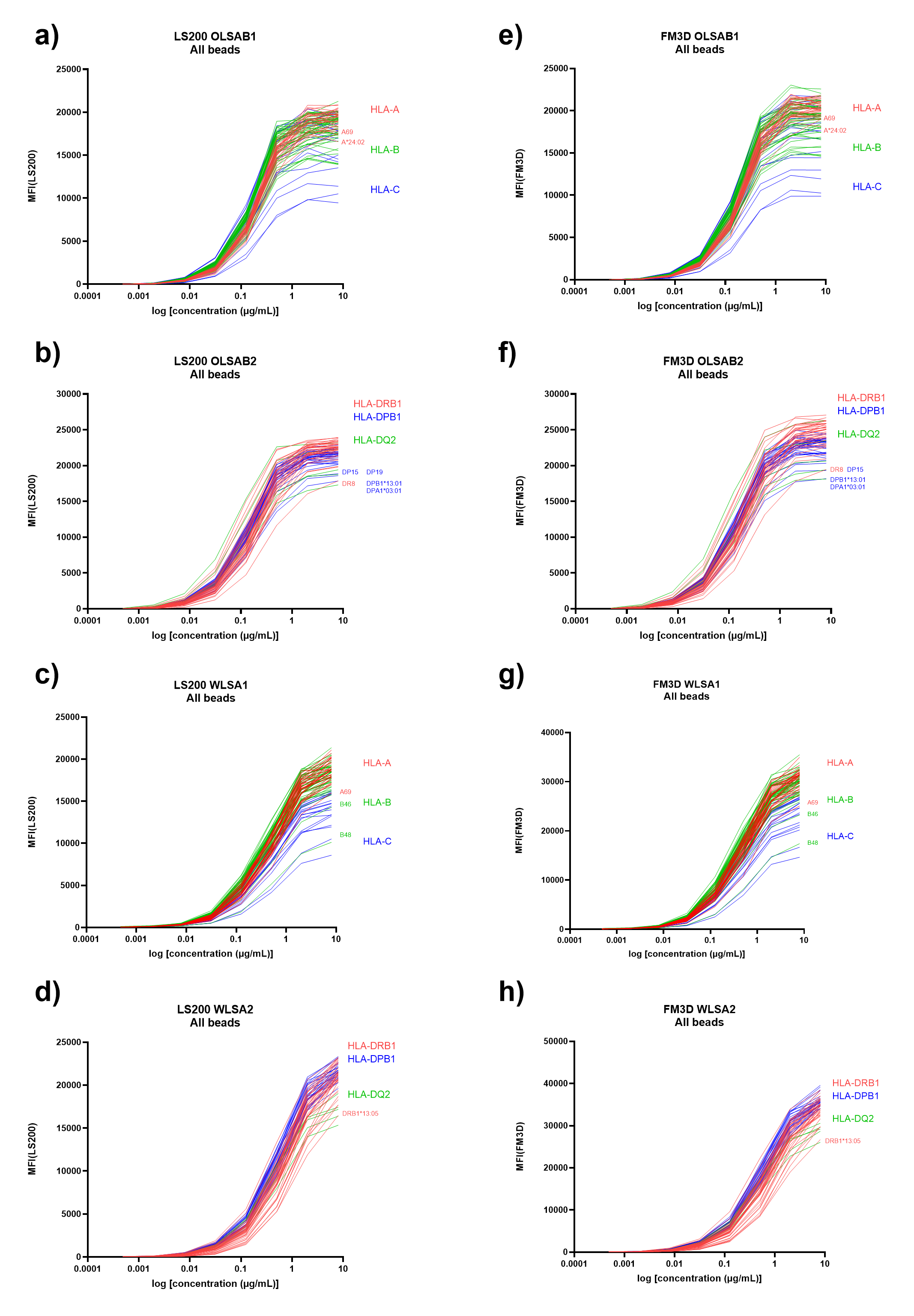

Supplement: Supplementary file 7 — Figure S6: Individual beads MFI for all conditions and outliers. [file TAN-107-e70731-s002.tif]

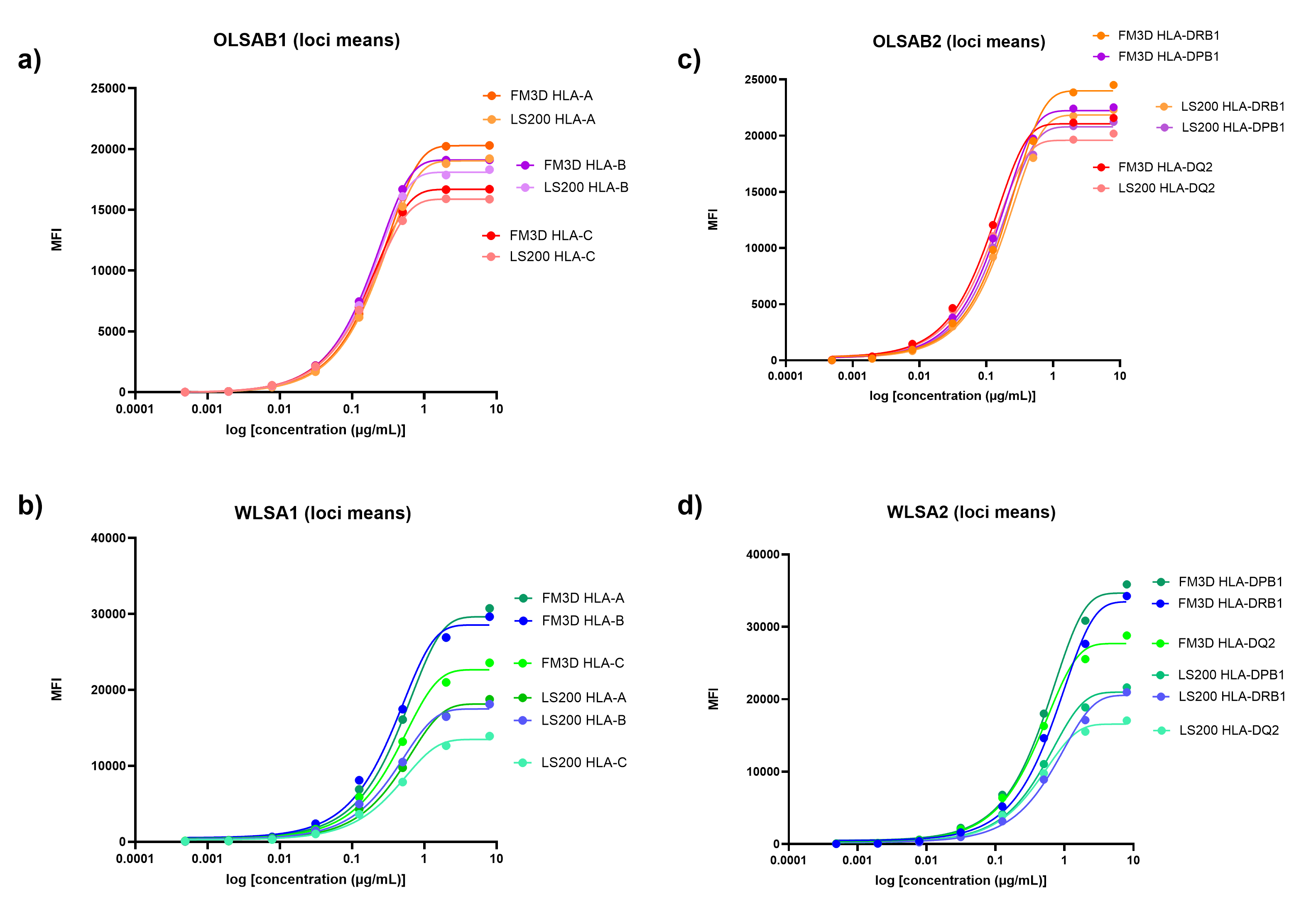

Supplement: Supplementary file 8 — Figure S7: Loci means MFI for all conditions. [file TAN-107-e70731-s001.tif]
