# Supplementary material for: Comparison of Labscan 200 and FlexMap 3D Luminex for Anti‐HLA Antibodies Monitoring
Source: HLA. 2026 May 5;107:e70731. doi: 10.1111/tan.70731 (PMC13144444; doi:10.1111/tan.70731)
Supplement: Supplementary file 9 — Data S1: Supporting Information. [file TAN-107-e70731-s005.docx]

**Supplemental Material**

More information for this article is available online. This includes additional comparisons between LS200 and FM3D Luminex: Boltzmann non-linear regressions for OLMIX (Figure S1), linear regressions for OLPRA1/2 (Figure S2) and bead variations for OLSAB1/2 and WLSA1/2 (Figure S3). Additionally, results from pure and 1:10 diluted sera are presented (Figure S4) and equivalences between LS200 and FM3D MFI are proposed (Figure S5). Last, sigmoids obtained from serial dilutions of anti-HLA monoclonal antibodies are presented, for each bead (Figure S6) and locus by locus (Figure S7, Table S1).
