# Supplementary material for: Comparison of Labscan 200 and FlexMap 3D Luminex for Anti‐HLA Antibodies Monitoring
Source: HLA. 2026 May 5;107:e70731. doi: 10.1111/tan.70731 (PMC13144444; doi:10.1111/tan.70731)
Supplement: Supplementary file 10 — Table S1: Threshold, EC50 and maximum MFI by locus for all conditions. [file TAN-107-e70731-s004.docx]

|  | SAB Class I | | | | | | | | | | | |
| --- | --- | --- | --- | --- | --- | --- | --- | --- | --- | --- | --- | --- |
|  | OLSAB1 | | | | | | WLSA1 | | | | | |
|  | LS200 | | | FM3D | | | LS200 | | | FM3D | | |
|  | HLA-A | HLA-B | HLA-C | HLA-A | HLA-B | HLA-C | HLA-A | HLA-B | HLA-C | HLA-A | HLA-B | HLA-C |
| Threshold (bottom + 3s) | 949 | 1580 | 1348 | 1200 | 1498 | 1407 | 1013 | 966 | 841 | 1235 | 1249 | 944 |
| EC50 | 0,21 | 0,16 | 0,15 | 0,22 | 0,17 | 0,15 | 0,49 | 0,39 | 0,41 | 0,50 | 0,38 | 0,43 |
| Maximum MFI | 19429 | 18396 | 16172 | 20699 | 19403 | 16972 | 20060 | 19223 | 14750 | 32815 | 31287 | 24956 |
|  | | | | | | | | | | | | |
|  | SAB Class II | | | | | | | | | | | |
|  | OLSAB2 | | | | | | WLSA2 | | | | | |
|  | LS200 | | | FM3D | | | LS200 | | | FM3D | | |
|  | HLA-DR | HLA-DP | HLA-DQ2 | HLA-DR | HLA-DP | HLA-DQ2 | HLA-DR | HLA-DP | HLA-DQ2 | HLA-DR | HLA-DP | HLA-DQ2 |
| Threshold (bottom + 3s) | 858 | 1148 | 1187 | 1023 | 1473 | 1317 | 622 | 785 | 737 | 726 | 1108 | 688 |
| EC50 | 0,17 | 0,13 | 0,10 | 0,18 | 0,13 | 0,10 | 0,73 | 0,52 | 0,41 | 0,75 | 0,54 | 0,43 |
| Maximum MFI | 22700 | 21544 | 20439 | 24997 | 22984 | 21862 | 22644 | 22857 | 17871 | 37195 | 38058 | 30134 |
